# Combining electromyography and Raman spectroscopy: optical EMG

**DOI:** 10.1002/mus.27937

**Published:** 2023-07-21

**Authors:** James J. P. Alix, Maria Plesia, Pamela J. Shaw, Richard J. Mead, John C. C. Day

**Affiliations:** ^1^ Sheffield Institute for Translational Neuroscience University of Sheffield Sheffield UK; ^2^ Cross‐Faculty Neuroscience Institute University of Sheffield Sheffield UK; ^3^ Interface Analysis Centre, School of Physics University of Bristol Bristol UK

**Keywords:** ALS, biomarker, EMG, muscle, optical EMG, Raman spectroscopy

## Abstract

**Introduction/Aims:**

Electromyography (EMG) remains a key component of the diagnostic work‐up for suspected neuromuscular disease, but it does not provide insight into the molecular composition of muscle which can provide diagnostic information. Raman spectroscopy is an emerging neuromuscular biomarker capable of generating highly specific, molecular fingerprints of tissue. Here, we present “optical EMG,” a combination of EMG and Raman spectroscopy, achieved using a single needle.

**Methods:**

An optical EMG needle was created to collect electrophysiological and Raman spectroscopic data during a single insertion. We tested functionality with in vivo recordings in the SOD1^G93A^ mouse model of amyotrophic lateral sclerosis (ALS), using both transgenic (*n* = 10) and non‐transgenic (NTg, *n* = 7) mice. Under anesthesia, compound muscle action potentials (CMAPs), spontaneous EMG activity and Raman spectra were recorded from both gastrocnemius muscles with the optical EMG needle. Standard concentric EMG needle recordings were also undertaken. Electrophysiological data were analyzed with standard univariate statistics, Raman data with both univariate and multivariate analyses.

**Results:**

A significant difference in CMAP amplitude was observed between SOD1^G93A^ and NTg mice with optical EMG and standard concentric needles (*p* = .015 and *p* = .011, respectively). Spontaneous EMG activity (positive sharp waves) was detected in transgenic SOD1^G93A^ mice only. Raman spectra demonstrated peaks associated with key muscle components. Significant differences in molecular composition between SOD1^G93A^ and NTg muscle were identified through the Raman spectra.

**Discussion:**

Optical EMG can provide standard electrophysiological data and molecular Raman data during a single needle insertion and represents a potential biomarker for neuromuscular disease.

## Graphical abstract

We present optical EMG, a combination of EMG and Raman spectroscopy. During a single needle insertion, both electrophysiological data, for example, positive sharp waves, and Raman spectra were collected. As Raman spectra provide data on the molecular composition of tissue, optical EMG can improve detection of disease.

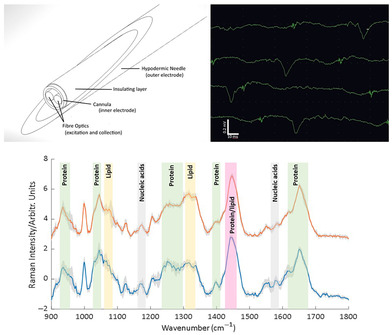

**Abbreviations**
ALS: amyotrophic lateral sclerosis
CMAP: compound muscle action potential
EMG: electromyography
NTg: non‐transgenic
PET: polytetrafluoroethylene
PSW: positive sharp wave
PTFE: polytetrafluoroethylene
SOD1: superoxide dismutase‐1

## 1 INTRODUCTION

Electromyography (EMG) records electrical activity arising from muscle. The standard needle electrode design is the concentric needle, in which a potential difference is taken between the outer shaft of the needle and an inner core.^1^ As the patient contracts the muscle of interest, the electrical activity of the recruited motor units is captured as motor unit action potentials which may be analyzed qualitatively by the expert physician, or quantitatively using dedicated software. While EMG provides rich detail on the structure and physiology of muscle through the detection of myofibre depolarisation, a wealth of information relating to disease remains undetected. This includes, for example, alterations in myofibre proteins, the presence of necrotic and regenerating fibers and infiltrating inflammatory cells. Detection of molecular information relating to these changes could provide additional diagnostic information in clinical practice and a biomarker for disease activity in clinical trials.

Raman spectroscopy is a form of vibrational spectroscopy that provides specific molecular information relating to the biochemical composition of the tissue. Thus, it could provide information complimentary to EMG. In Raman spectroscopy, light of a single wavelength undergoes an interaction with the electron cloud of molecules within the matter under study, in this context, muscle. The interaction is not stable and the light is almost instantly released or “scattered.” Most scattered photons have the same energy as the incident light, but a small proportion have their energy, and therefore wavelength, changed. This energy change, which is called Raman scattering, is dependent upon the molecular bonds the light has encountered. Thus, by analyzing the wavelength spectrum of Raman scattered light, a molecular “fingerprint” of the tissue can be obtained.

While Raman scattering is a relatively weak phenomenon (only around one in 10^6^–10^9^ photons undergo Raman scattering), technological advancements have resulted in increasingly sensitive detection of the Raman signal.^2^ As a result, Raman spectroscopy is under intense investigation for the real‐time diagnosis of many different human diseases, with promising results observed for multiple cancers^3^ and, with relevance to the present study, neurological diseases.^4^, ^5^, ^6^, ^7^, ^8^ Using a fiber optic Raman spectroscopy probe in which optical fibers were passed down a standard hypodermic needle, we developed an in vivo preclinical methodology capable of identifying neurogenic and myogenic disease^5^ and monitoring disease state.^9^ Using the same probe, we have also demonstrated the diagnostic potential of the technique to identify muscle disease in human biopsy specimens.^10^, ^11^

Herein, we have developed a combined Raman spectroscopy/EMG needle to undertake what we term “optical EMG.” Our aim was to assess the basic functionality of the needle and demonstrate the principle of undertaking both measurements during the same insertion.

## 2 METHODS

### 2.1 Optical EMG needle

The starting point for the design of the optical EMG needle was a subcutaneous Raman needle probe,^12^ which we have previously used to collect Raman spectra from both murine and human muscle.^5^, ^9^, ^10^, ^11^ In the present device, two optical fibers were housed within a 23‐gauge stainless steel canula. These fibers respectively convey the laser excitation light and the Raman scattered light to and from the sample for spectroscopic measurements at the tip of the canula. The cannula was passed down the center of a conventional 21‐gauge hypodermic needle. During insertion, the cannula was held retracted (i.e., away from the needle tip) and the needle inserted into the target tissue. Once in place the cannula was advanced such that it just passed the end of the needle, as previously described.^12^ To enable EMG measurements, a thin sleeve of polyethylene terephthalate (PET) heat shrink tubing (Nordson Medical, Westlake, Ohio, USA) was placed over the cannula to electrically insulate it from the hypodermic needle. The distal tip of the cannula was, however, exposed so that when deployed, the end face made electrical contact with the tissue. In this way, the cannula served as the inner core of a conventional concentric EMG needle, while the outer hypodermic needle served as the corresponding outer electrode. Figure S1 shows the arrangement of the distal tip in the deployed position. Simple spring clips were used to make electrical contact to the proximal ends of the canula and hypodermic needle and these were attached to standard EMG equipment (see below).

For Raman data collection, the needle was connected to an 830 nm semiconductor laser (Innovative Photonics Solutions, Plainsboro Township, New Jersey, USA).^13^ In‐line filters were used to reduce the inelastically scattered light and fluorescence. These comprised a bandpass filter on the excitation delivery fiber and a long‐pass filter on the collection fiber (Semrock Inc., Rochester, New York, USA). All fibers were low OH, with a 105 μm silica core of numerical aperture 0.22. The optic EMG needle was optically matched to the spectrometer (Raman Explorer Spectrograph, Headwall Photonics Inc., Bolton, Massachusetts, USA and iDus 420BR‐DD CCD camera, Andor Technology, Ltd, Belfast, Northern Ireland, UK). The Raman signal, which samples from a tissue volume of approximately 0.25 mm^3^, was recorded through a 40 s exposure consisting of 10 × 4 second epochs which were averaged. Spectra from polytetrafluoroethylene (PTFE) were acquired for wavenumber calibration.

For electrophysiological data collection, the optical EMG needle was connected to a Dantec Keypoint Focus EMG system (Natus Medical Inc., Middleton, Wisconsin, USA), with standard filter settings (20 Hz–10 kHz). Compound muscle action potential (CMAP) recordings were also made using a commercial concentric EMG needle (Ambu Neuroline, Ballerup, Denmark, 30G).

### 2.2 In vivo testing

All mouse experiments were carried out in accordance with the Animals (Scientific Procedures) Act 1986, under a UK Home Office project license (number 70/8587). The project was approved by the University of Sheffield Animal Welfare and Ethical Review Body (AWERB). Mice were housed in a standard facility (12‐h light/dark cycle, room temperature 21°C) and cared for in accordance with the Home Office Code of Practice for Housing and Care of Animals Used in Scientific Procedures. The ARRIVE guidelines were followed in the conduct of this work.^14^

Transgenic C57BL/6J‐Tg (SOD1^G93A^)1Gur/J mice, a model of amyotrophic lateral sclerosis (ALS), were used; these were originally obtained from Jackson Laboratories. Hemizygous transgenic males were backcrossed to C57BL/6 females (Harlan UK, C57BL/6 J OlaHsd substrain) in our facility for >20 generations.^15^ Hemizygous females were used for experiments, with non‐transgenic (NTg) females used as controls. Transgenic SOD1^G93A^ mice were identified through polymerase chain reaction amplification of genomic DNA extracted from ear clips.^16^ These mice are extremely well characterized and manifest similar electrophysiological/histological abnormalities to those observed in ALS, including reduced CMAP amplitudes, motor unit counts, spontaneous EMG activity and grouped muscle fiber atrophy.^15^, ^17^, ^18^, ^19^, ^20^, ^21^ This allowed us to select an age at which the hindlimb muscles manifest relevant electrophysiological and histological changes. We elected to undertake recordings at 90‐days of age, which we have previously utilized for in vivo testing of a Raman‐only fiber optic probe.^5^ A total of 17 mice (*n* = 10 SOD1^G93A^ and *n* = 7 NTg) were used in the study.

For optical EMG recordings, mice were anesthetized using 2% isoflurane and placed on a heat pad to maintain body temperature. The hindlimbs were shaved and the optical EMG needle inserted into both gastrocnemius muscles. CMAPs were first elicited using a 0.1 ms duration stimulus applied at the sciatic notch. Stimulation intensity was adjusted to obtain a supra‐maximal response and baseline‐to‐negative peak amplitude was recorded. Raman data were then recorded. In *n* = 8 SOD1 mice and *n* = 7 NTg mice, repeat CMAPs were then recorded post‐Raman in one leg.

For measurements using commercial concentric EMG needles, recordings were made using the same methodology shortly after the optical EMG recording. Mice were humanely sacrificed after recording completion.

### 2.3 Data analysis

For electrophysiological data, univariate statistical analysis was performed using GraphPad Prism (version 9, Dotmatics, Boston, Massachusetts, USA). Differences in CMAP amplitude between SOD1^G93A^ and NTg mice (for both the optical EMG needle and commercial concentric EMG needle) were analyzed using nested *t*‐tests (CMAPs nested within mice, since each mouse had a CMAP taken from each leg). Assessments of pre‐ vs. post‐Raman CMAP amplitudes (taken with the optical EMG needle) were made using a paired *t*‐test. Bland–Altman plots (Figure S2) were produced using data from the optical EMG and concentric EMG needles.

Raman data are typically analyzed with complex multivariate techniques, often termed “chemometrics.”^22^ Herein, Raman spectral analysis was performed using custom code in MATLAB (MATLAB R2021b, The MathWorks, Natick, Massachusetts, USA). Raw spectra were first interpolated to integer wavenumber spacings between 900 and 1800 cm^−1^. Spectral windowing between 900 cm^−1^ and 1800 cm^−1^ was performed to capture information within the “fingerprint” region where biological information is contained.^23^ Below this window spectra were obscured by a silica‐related Raman signal generated within the optical fibers; above this window spectra consist only of noise. Background subtraction was undertaken using the rubber band algorithm,^24^ followed by Savitzky–Golay smoothing (second‐order filter, five data point window width) and vector normalization. As a first analysis step, the difference between the means of the two groups was plotted (SOD1^G93A^ *minus* NTg). Next, peak ratios were chosen to demonstrate example differences in the molecular composition of the muscle. Group differences were examined using nested *t*‐tests. An example of a more complex multivariate analysis at the level of the whole spectrum is given in the Supplementary file.

## 3 RESULTS

**FIGURE 1:**
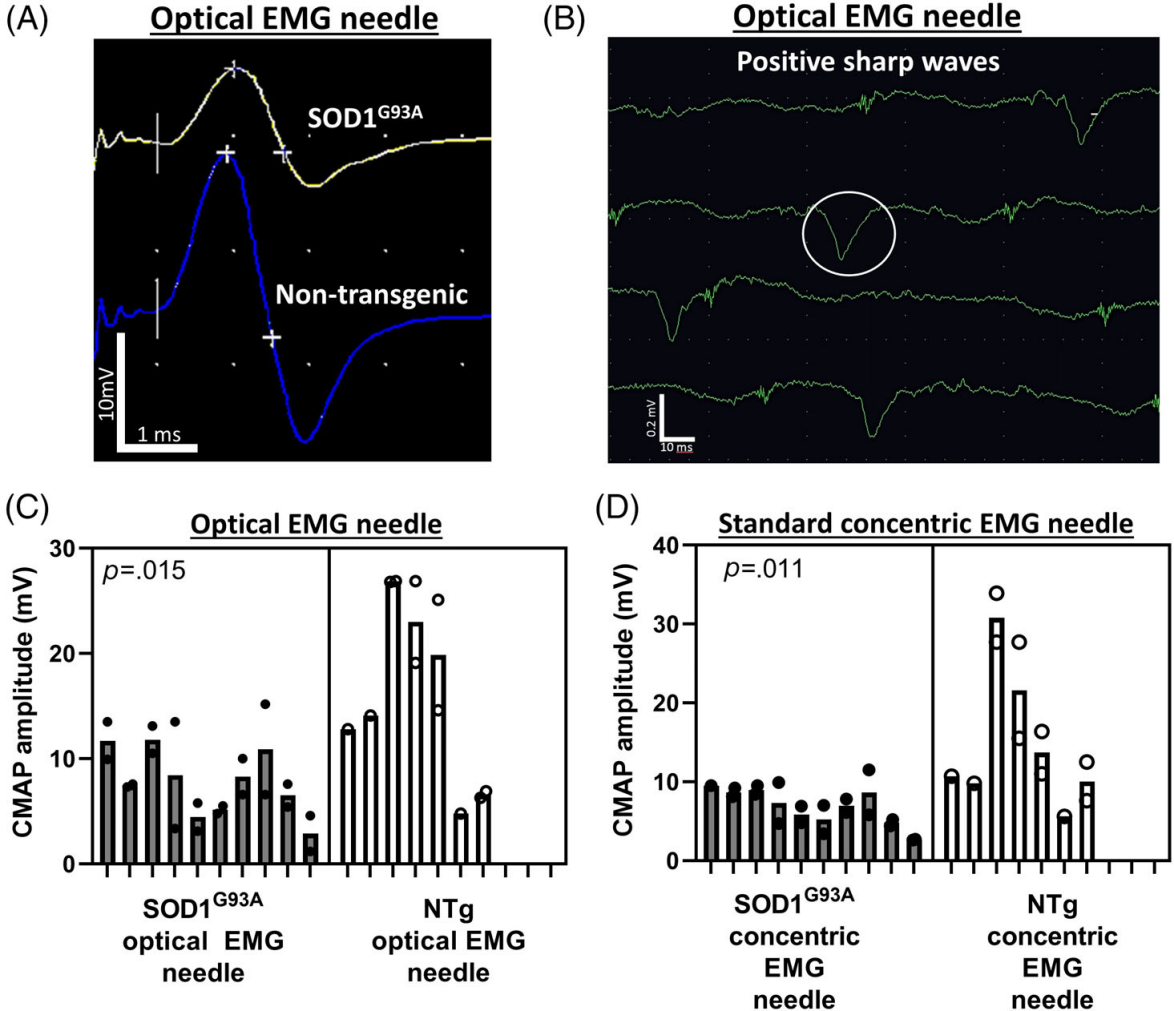
Optical EMG: electrophysiological data. (A) CMAP waveforms from both SOD1^G93A^ and NTg mice using the optical EMG needle. (B) Spontaneous EMG activity (PSWs; example circled) from the optical EMG needle. Note that the PSWs here have an irregular inter‐wave interval as the run was coming to an end. See supplemental information for further examples. (C) Comparison of CMAP amplitude in NTg and SOD1^G93A^ mice using the optical EMG needle. (D) Comparison of CMAP amplitude in NTg and SOD1^G93A^ mice using a standard concentric EMG needle.

Using the optical EMG needle, gastrocnemius CMAPs could be recorded following stimulation of the sciatic nerve (Figure 1A). Significant differences in CMAP amplitudes recorded from SOD1^G93A^ and NTg mice were observed for both the optical EMG probe and a standard concentric EMG needle (Figure 1C,D, respectively). CMAP amplitudes between the optical EMG probe and standard EMG needle were similar (Figure S2). In addition, spontaneous EMG activity in the form of positive sharp waves (PSWs) was recorded using the optical EMG needle (Figure 1B). Further examples of EMG activity can be found in Figure S3 and in the accompanying video file.

**FIGURE 2:**
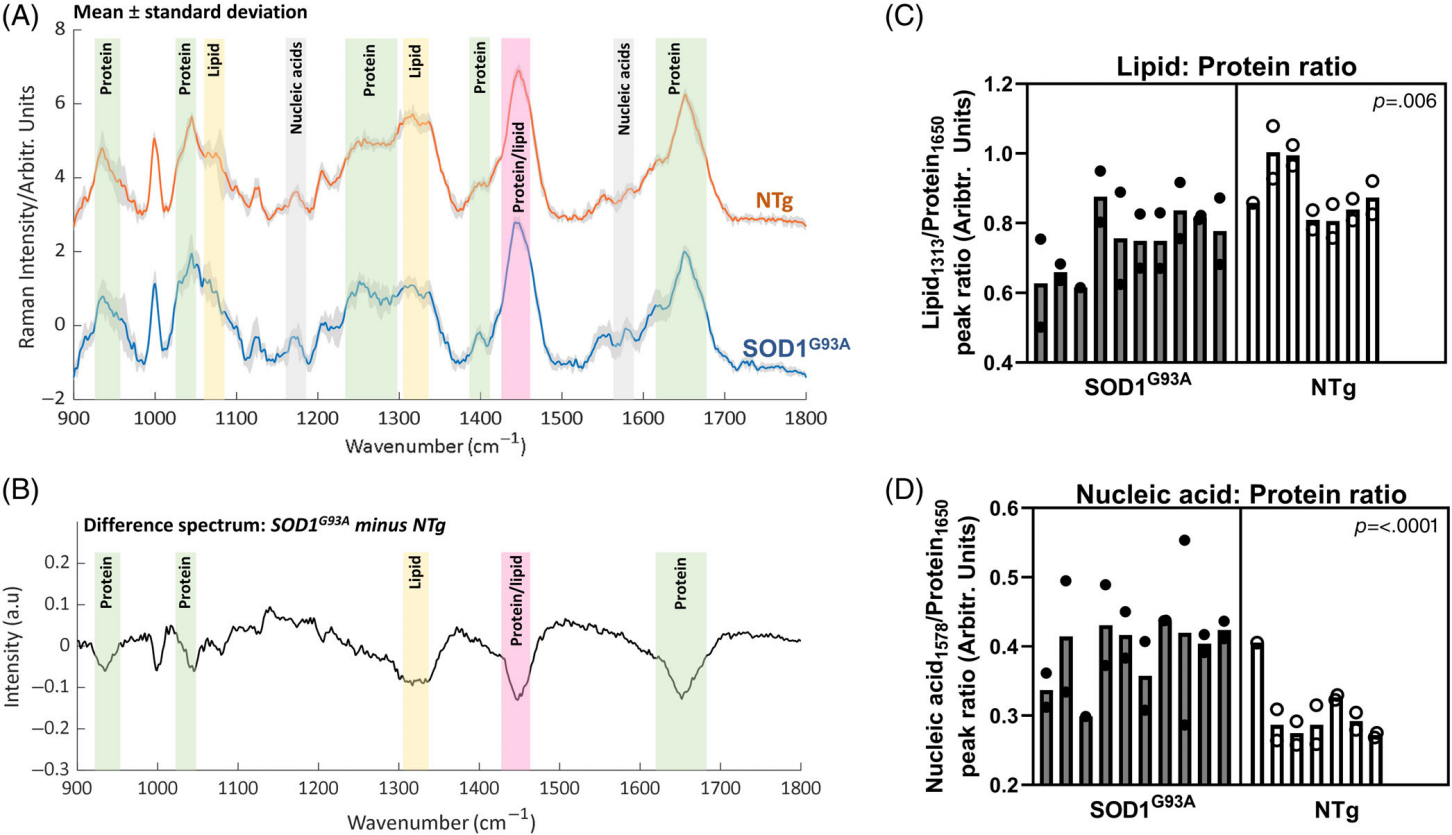
Optical EMG: Raman spectroscopy data. (A) Mean Raman spectra (± SD) for NTg (top, orange) and SOD1^G93A^ mice (bottom, blue), both at 90 days. The two spectra have been arbitrarily offset on the *y*‐axis so that the two plots can be clearly visualized. Basic biochemical assignments are shown by colored bars. (B) Difference of the mean spectrum (SOD1^G93A^ minus NTg). This highlights the spectral regions at which the two groups differ the most. (C, D) Peak ratios exploring differences in the molecular composition of SOD1^G93A^ and NTg muscle.

Immediately after the collection of electrophysiological data, with the needle remaining in situ, Raman spectra were acquired (Figure 2A). Simple biochemical peak assignments are shown; specific wavenumbers and their assignments are detailed in Figure S4 and Table S1. Difference spectra (mean of SOD1^G93A^ minus mean of NTg) demonstrated increased concentrations of peaks relating to protein structure in NTg mice, for example, 935, 1045, 1448 and 1652 cm^−1^ (Figure 2B). The ratio of specific peaks within the Raman spectrum can also be used to investigate changes in molecular composition. Two such ratios, relating to lipid/protein content and nucleic acid/protein content, are shown in Figure 2 and demonstrate differences between SOD1^G93A^ and NTg mice. More complex chemometric analyses can also be undertaken to characterize the entire molecular fingerprint provided by the spectra. An example of this, with a schematic overview of the method, is provided in Figures S5 and S6.

**FIGURE 3:**
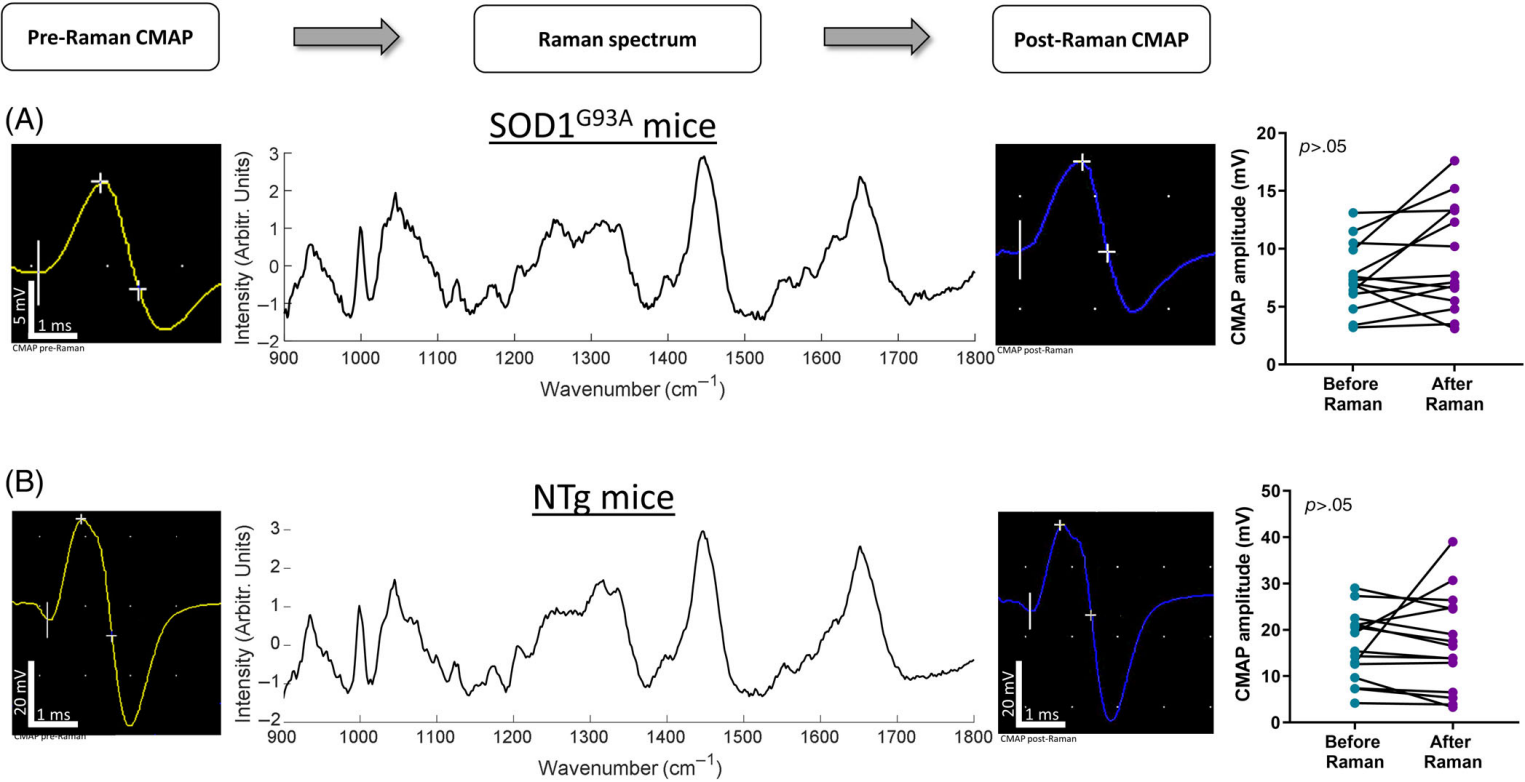
In vivo intra‐muscular Raman spectroscopy does not alter CMAP amplitude. (A) Example CMAPs and Raman spectra from SOD1^G93A^ mice, recorded using the optical EMG needle. No significant difference was seen between the pre‐ and post‐Raman CMAP amplitudes. For convenience, right and left leg data are shown on the same plot. (B) Example CMAPs and Raman spectra from NTg mice. No significant difference was seen between the pre‐ and post‐Raman CMAP amplitudes. As above, right and left leg data are shown on the same plot.

After recording the Raman spectra and prior to needle removal, we repeated the CMAP measurements (Figure 3). No significant change in CMAP amplitude was seen after Raman spectra were acquired in either SOD1^G93A^ or NTg mice.

## 4 DISCUSSION

In this work, we have demonstrated the functionality of a combined EMG/Raman spectroscopy needle, a technique we were refer to as “optical EMG.” The results demonstrate that the probe can record high quality electrophysiological and Raman data in vivo. The work provides the first step toward developing optical EMG as a translational biomarker of muscle health.

The biochemical information provided by Raman spectroscopy is complimentary to the electrophysiological information provided by EMG. On a practical level, concomitant EMG can confirm that the Raman probe is in the muscle of interest. Other techniques such as ultrasound can also offer this; however, real‐time analysis of EMG activity could also be used to target the Raman probe to electrically abnormal (and normal) areas of muscle. While not yet investigated, it is intuitive to hypothesize that this may increase the likelihood of obtaining clinically useful molecular information. However, the sensitivity of Raman spectroscopy to non‐excitable muscle components (fats, proteins, connective tissue), means that Raman may have the potential to provide clinical useful information in “EMG normal” muscle. Further testing through in vivo studies in human subjects will help clarify the best way to deliver Raman to muscles of interest. Such studies would also allow for correlation between Raman spectroscopy and other markers of muscle function/pathology, for example, patient symptom scales and imaging modalities.

The method for detecting muscle membrane depolarisation with the optical EMG probe is similar to that of a standard concentric EMG needle. In a standard EMG needle, a potential difference is taken between an outer needle cannula typically made of steel (acting as a “reference”) and an inner silver or platinum wire called the core (which acts as the active electrode).^25^ The core of the needle is embedded within an insulating material. In the present study, we used a commerically available 30G concentric EMG needle, which has a needle diameter of 0.3 mm and a bevel which exposes the central wire as an oblique, elliptical surface ground to an angle of around 15 degrees. The resulting recording area is documented as 0.02 mm^2^.^26^ Our optical EMG needle cannula was a standard 21G hypodermic needle, with an outer cannula diameter of 0.819 mm, inner diameter of 0.514 mm, and bevel angle of 12 degrees. For the demonstration of proof of concept, we did not set out to replicate the electrophysiological characteristics of a standard concentric EMG needle in our optical EMG needle. Thus, it is inevitable that there will be differences in, for example, the pickup area of the two needles. Despite this, in our comparison of CMAP amplitudes, no consistent difference was seen between the optical EMG needle and concentric needle electrode, and our data are similar to those from previous studies.^27^ Thus, we have a good starting point for further development. However, we fully acknowledge that CMAP amplitudes are not the parameter of interest for needle EMG. Further studies, either on mice as the anesthesia is lightened to permit motor unit recruitment, or in human subjects, will be required to understand if the optical EMG needle can collect clinically useful motor unit potential information. At present, work is ongoing for the design of a smaller optical EMG needle that will be more closely aligned with standard EMG needles.

The optical EMG needle produced Raman data comparable to that previously obtained with a single Raman‐only probe^5^; this was expected as pathways for both the incident and scattered light were unchanged. Prominent protein peaks were observed, likely relating to muscle proteins such as myosin and actin.^28^, ^29^ Differences in the lipid:protein ratio are in keeping with prior studies in the SOD1^G93A^ model, which have shown increased lipid catabolism and inhibition of fat deposition within muscle fibers.^30^, ^31^ Changes relating to nucleic acid content are likely to be complex but the infiltration of immune cells and changes in the “inflammasome” that have been reported in both murine SOD1 models and patients may be a contributor.^32^, ^33^

In our previous in vivo preclinical work with a Raman‐only probe, we were able to discriminate between neurogenic and myopathic pathology, and different stages of disease.^5^, ^9^ Ex vivo human muscle biopsy analyses with the same Raman‐only probe were also promising in identifying muscle pathology, including different types of muscle disease.^10^, ^11^ Thus, while we did not study different types of muscle pathology on this occasion, we anticipate that optical EMG should be capable of providing similar information, enabling discrimination between different neuromuscular disorders and different stages of disease. Thus, optical EMG could provide disease specific data, which at present is only obtained through a more invasive muscle biopsy. A key advantage of optical EMG would be the potential to examine several areas within a muscle, as well as multiple muscles. This may increase the likelihood of obtaining disease specific information, which can sometimes be missed on muscle biopsies.^34^, ^35^, ^36^

In the present study, we observed no significant difference between pre‐ and post‐Raman CMAP amplitudes. This indicates that the thermal energy from the laser did not have any deleterious effects on the ability of the muscle fibers to depolarise in response to supra‐maximal nerve stimulation. These observations are in keeping with our previous work which demonstrated no motor impairment after in vivo Raman recordings and resolution of mild T2 signal changes on 7T MRI by 2‐wk post‐procedure.^5^ Multiple groups have performed in vivo Raman measurements on a variety of tissues with no evidence of significant tissue injury, highlighting the potential of Raman spectroscopy as a safe technique for tissue analysis.^37^, ^38^, ^39^, ^40^, ^41^, ^42^

In conclusion, we present the technique of optical EMG, a novel combination of electrophysiology and Raman spectroscopy. As proof of concept, we demonstrate that optical EMG can provide sensitive, quantitative measures of disease using the SOD1^G93A^ model of ALS. The work illustrates the potential of this new technique for the detection and diagnosis of neuromuscular disease.

## AUTHOR CONTRIBUTIONS

**James J.P. Alix:** Conceptualization; methodology; formal analysis; data curation; resources; project administration; writing – original draft; funding acquisition; investigation. **Maria Plesia:** Investigation; writing – review and editing. **Pamela J. Shaw:** Funding acquisition; writing – review and editing. **Richard J. Mead:** Writing – review and editing; funding acquisition; resources. **John C. C. Day:** Conceptualization; methodology; investigation; funding acquisition; writing – review and editing.

## FUNDING INFORMATION

The work was support by a Medical Research Council Confidence in Concept award (J.J.P.A., J.C.D., R.J.M., P.J.S., MC_PC_15034). P.J.S. is supported as a National Institute for Health Research (NIHR) Senior Investigator (NF‐SI‐0617‐10077) and by the NIHR Sheffield Biomedical Research Centre (IS‐BRC‐1215‐20017).

## CONFLICT OF INTEREST STATEMENT

J.J.P.A. and J.C.C.D. are the joint authors of a UK patent application concerning the described technology, filed September 2022, number 2214072.7.

## ETHICS STATEMENT

We confirm that we have read the Journal's position on issues involved in ethical publication and affirm that this report is consistent with those guidelines.

## Supporting information


**Video S1:** Spontaneous EMG activity recorded with the optical EMG needle. Fibrillation potentials are followed by a complex repetitive discharge.

**Data S1.** Supporting Information.

## Data Availability

The data that support the findings of this study are available from the corresponding author upon reasonable request.
