# Supplementary material for: Combining electromyography and Raman spectroscopy: optical EMG
Source: Muscle Nerve. 2023 Jul 21;68(4):464–70. doi: 10.1002/mus.27937 (PMC10952815; doi:10.1002/mus.27937)
Supplement: Supplementary file 2 — Data S1. Supporting Information. [file MUS-68-464-s001.docx]

**Supplementary methods**

To analyse molecular changes in more detail, and quantify such differences, Raman data are analysed with multivariate techniques. Here, we utilise a form of matrix factorisation termed bounded structured simplex matrix factorisation (BSSMF).^1^ In this, the dominant spectral patterns within the data are derived in an unsupervised manner i.e. without taking into consideration the disease/healthy categories. To do this, BSSMF approximates the original data, ***X***, as the product of two matrices

$$\boldsymbol{X}\approx\boldsymbol{WH}$$

where the data in ***W*** represent the dominant spectral patterns and the data in ***H*** represent the weight, or importance, of each pattern to each sample.

Analyses were performed using custom code in MATLAB. To define the number of spectral patterns to be derived from the original data we calculated the root mean square residual between the left and right legs of healthy NTg mice. Since no biological difference is expected between the legs of healthy mice, the residual can be used to define the threshold the BSSMF approximation. The root mean square residual between the original and approximated data was calculated for different numbers of spectral patterns and the number of patterns first reaching the pre-defined threshold was selected for ensuing analyses. In this instance, 3 patterns were required. For each pattern, every sample is allocated a weight (in matrix ***H***) which represents the importance of that pattern to that sample.

Next, principal component analysis (PCA) was applied to the weights (matrix ***H***) and the direction of maximum variance (i.e. principal component 1) calculated. The principal component coefficients were then used to produce a linear combination of the three patterns. This reduces the three BSSMF patterns down to two, one which dominates in SOD1 ^G93A^ and another which dominates in NTg. In addition, rather than there being three weight scores for each pattern, there is now just one weight score, and this single score represents the importance of the two new patterns to a single sample. For these data, negative scores were associated with SOD1 ^G93A^ and positive scores with NTg. The descriptive term for this analysis would be *bounded simplex-structured matrix factorization fed principal component analysis* *for Raman data,* which we call ‘BPR’ for convenience. See Alix et al., for further details on this approach to analysing Raman data.^2^ The BPR scores were analysed with a nested t-test (scores nested within each mouse) on GraphPad Prism.

**Supplementary results**

| **Wavenumbers (cm^-1^)** | **Assignment** |
| --- | --- |
| 934/5/6 | Protein (α-helix)^3,4^ |
| 999/1000 | Phenylalanine^5^ |
| 1041-1052 | Proteins^6,7^ |
| 1076 | C-C stretch lipids^8^ |
| 1125 | Proteins (collagen), lipids^9,8^ |
| 1140 | Collagen^10^ |
| 1175 | Nucleic acids^11^ |
| 1205-1215 | Tyrosine, phenylalanine^4,7^ |
| 1258 | Amide III (protein)^5^ |
| 1313 | CH_3_CH_2_ twisting mode of lipids^12^ |
| 1335 | CH_3_CH_2_ wagging mode of collagen and DNA – purine bases^12^ |
| 1230-1300 | Proteins^12,5^ |
| 1315-1340 | CH_2_CH_3_ deformation, proteins/lipids^13^ |
| 1400 | CH_3_ bending vibration of proteins^14^ |
| 1442/7 | CH modes (CH_2_ and CH_3_ deformations: bending & scissoring) in proteins/lipids^12,15^ |
| 1578 | Pyrimidine ring, nucleic acids^12^ |
| 1651/2 | Proteins (amide I)^4,16,17^ |

**Supplementary table 1.**

**Tentative peak assignments for the wavenumber labels found on the average Raman spectra and the BPR derived spectra.**


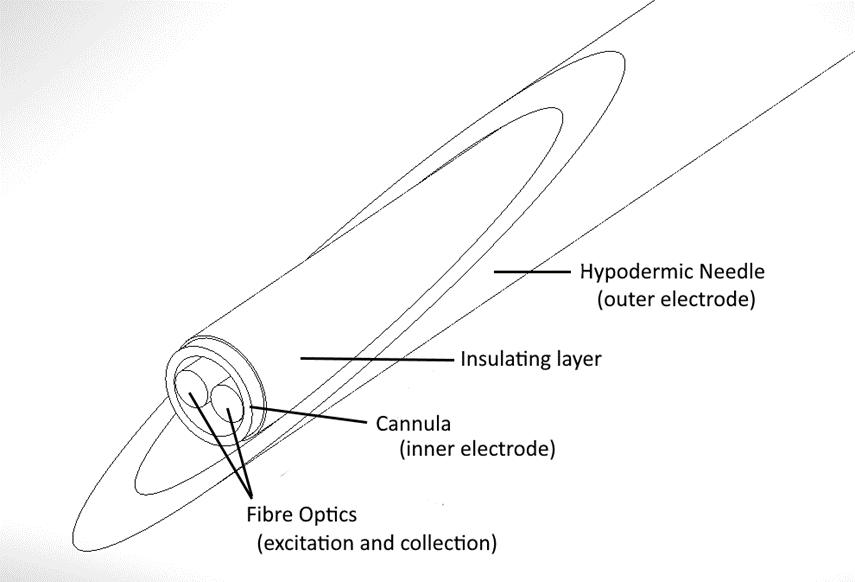


**Supplemental figure 1.**

**Schematic of the distal tip of the optical EMG probe.**


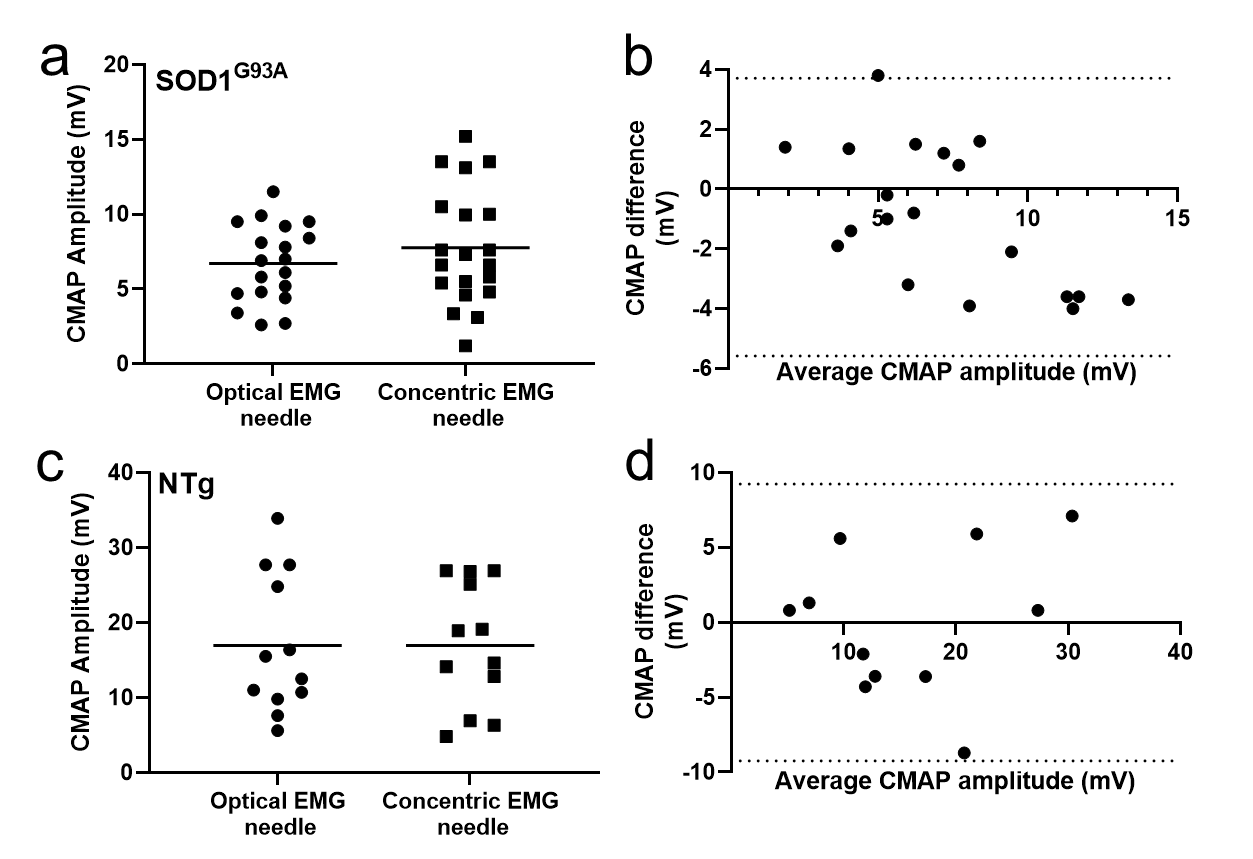
**Supplementary figure 2.**

**Comparison of optical EMG probe and standard EMG needle CMAP amplitudes.**

a). CMAP amplitudes obtained in SOD1^G93A^ mice with the optical EMG needle and standard concentric EMG needle.

b). Bland-Altman plot (optical EMG needle CMAP amplitude minus concentric needle CMAP amplitude). Bias = 0.93.

c). CMAP amplitudes obtained in NTg mice.

d). Bland-Altman plot (optical EMG needle CMAP amplitude minus concentric needle CMAP amplitude). Bias = -2.961e-016


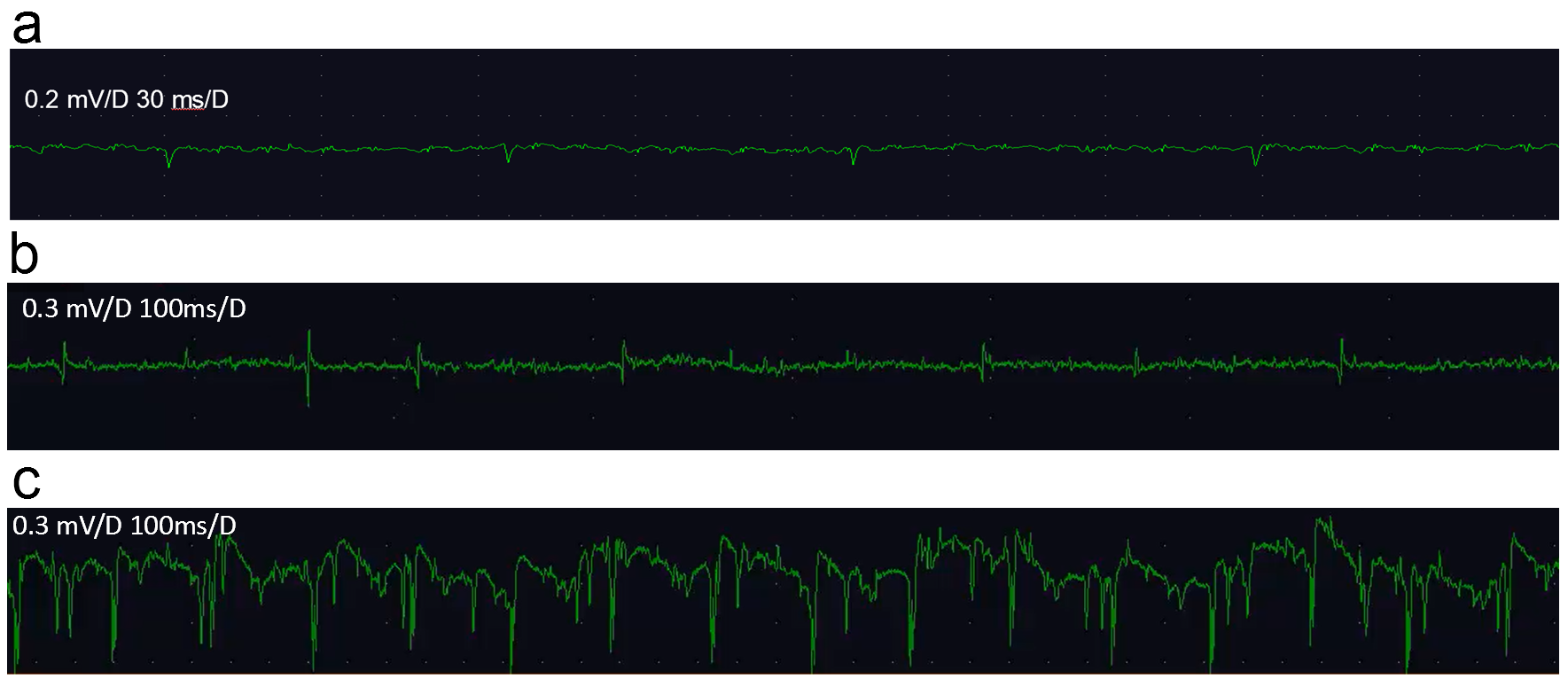


**Supplemental figure 3.**

**Optical EMG: EMG waveforms.**

a). Regular positive sharp waves.

b). Fibrillation potentials.

c). Complex repetitive discharge.


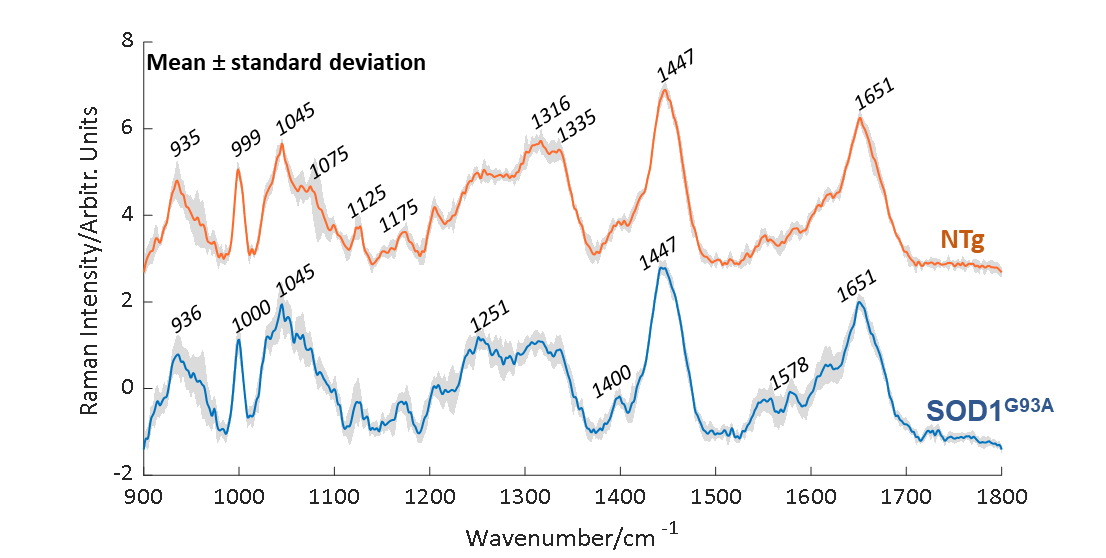


**Supplementary figure 4.**

**Mean (± standard deviation) spectra for the two groups with prominent peaks identified.**

**
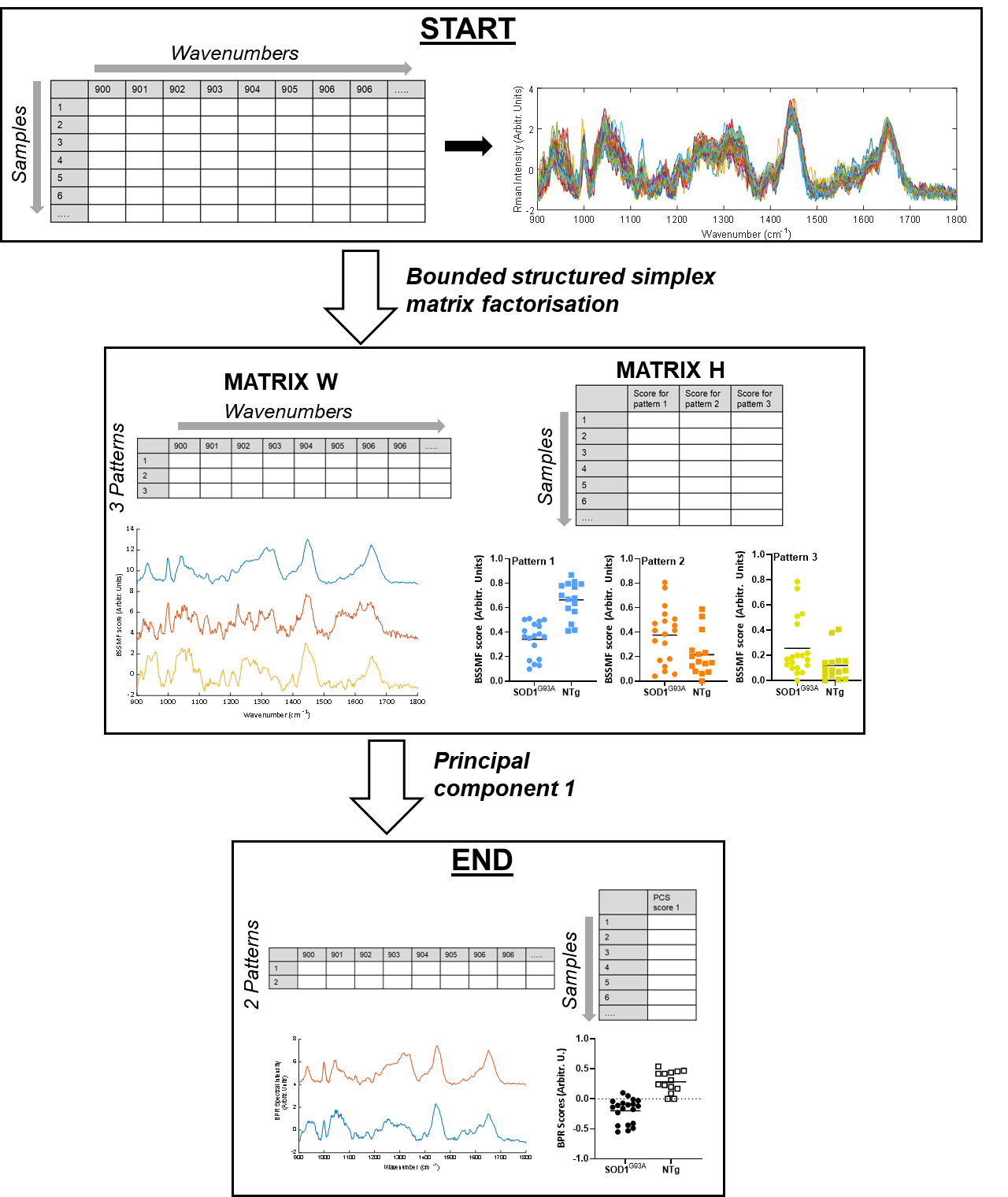
**

**Supplementary figure 5.**

**The multivariate BPR analysis.**

Data first comprise all the samples and all the spectra. Matrix factorisation is then applied to find the dominant spectral patterns within the data (matrix W); herein, three patterns were found. For each pattern, each sample is assigned a ‘score’ denoting the importance of a given pattern to a given sample (matrix H). Next, PCA is applied. This reduces the three patterns down to two patterns: one for disease, one for healthy. A further advantage is that now there is just one score per sample. This type of analysis means that the whole spectrum is analysed, rather than just specific peaks, as shown in the main manuscript. Thus, a more complete biochemical fingerprint is achieved for each group.

**
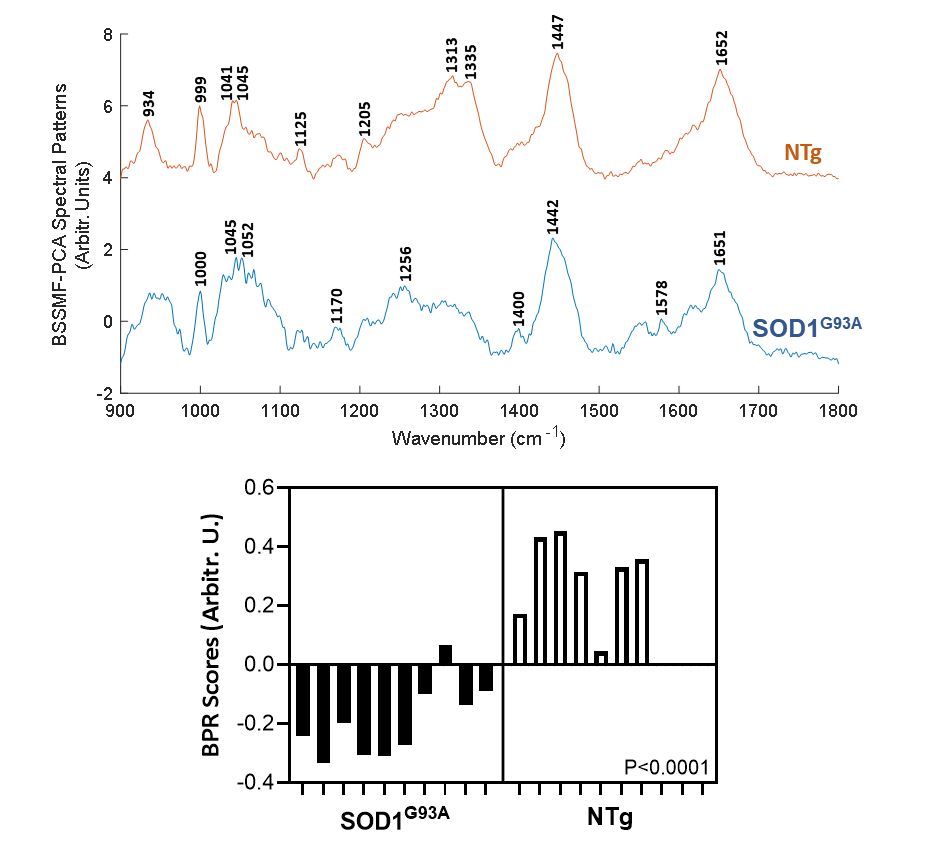
**

**Supplemental figure 6.**

**The final BPR patterns and scores resulting from the analysis shown in supplemental figure 5.**

**References**

1. Thanh OV, Gillis N, Lecron F. Bounded Simplex-Structured Matrix Factorization. 2022 23-27 May 2022. p 9062-9066.

2. Alix JJP, Verber NS, Schooling CN, Kadirkamanathan V, Turner MR, Malaspina A, Day JCC, Shaw PJ. Label-free fibre optic Raman spectroscopy with bounded simplex-structured matrix factorization for the serial study of serum in amyotrophic lateral sclerosis. Analyst 2022.

3. Bonnier F, Byrne HJ. Understanding the molecular information contained in principal component analysis of vibrational spectra of biological systems. Analyst 2012;137(2):322-332.

4. Stone N, Kendall C, Smith J, Crow P, Barr H. Raman spectroscopy for identification of epithelial cancers. Faraday Discuss 2004;126:141-157; discussion 169-183.

5. Kirkby CJ, Gala de Pablo J, Tinkler-Hundal E, Wood HM, Evans SD, West NP. Developing a Raman spectroscopy-based tool to stratify patient response to pre-operative radiotherapy in rectal cancer. Analyst 2021;146(2):581-589.

6. Zhu G, Zhu X, Fan Q, Wan X. Raman spectra of amino acids and their aqueous solutions. Spectrochim Acta A Mol Biomol Spectrosc 2011;78(3):1187-1195.

7. De Gelder J, De Gussem K, Vandenabeele P, Moens L. Reference database of Raman spectra of biological molecules. Journal of Raman Spectroscopy 2007;38(9):1133-1147.

8. Huang N, Short M, Zhao J, Wang H, Lui H, Korbelik M, Zeng H. Full range characterization of the Raman spectra of organs in a murine model. Opt Express 2011;19(23):22892-22909.

9. Huang Z, McWilliams A, Lui H, McLean DI, Lam S, Zeng H. Near-infrared Raman spectroscopy for optical diagnosis of lung cancer. Int J Cancer 2003;107(6):1047-1052.

10. Yorucu C, Lau K, Mittar S, Green NH, Raza A, Rehman IU, MacNeil S. Raman spectroscopy detects melanoma and the tissue surrounding melanoma using tissue-engineered melanoma models. Appl Spectrosc Rev 2016;51(4):243-257.

11. Talari ACS, Movasaghi Z, Rehman S, Rehman Iu. Raman Spectroscopy of Biological Tissues. Applied Spectroscopy Reviews 2015;50(1):46-111.

12. Stone N, Kendall C, Shepherd N, Crow P, Barr H. Near-infrared Raman spectroscopy for the classification of epithelial pre-cancers and cancers. Journal of Raman Spectroscopy 2002;33(7):564-573.

13. Ruiz-Chica AJ, Medina MA, Sánchez-Jiménez F, Ramírez FJ. Characterization by Raman spectroscopy of conformational changes on guanine–cytosine and adenine–thymine oligonucleotides induced by aminooxy analogues of spermidine. Journal of Raman Spectroscopy 2004;35(2):93-100.

14. Duraipandian S, Zheng W, Ng J, Low JJ, Ilancheran A, Huang Z. In vivo diagnosis of cervical precancer using Raman spectroscopy and genetic algorithm techniques. Analyst 2011;136(20):4328-4336.

15. Silveira L, Jr., Silveira FL, Bodanese B, Zangaro RA, Pacheco MT. Discriminating model for diagnosis of basal cell carcinoma and melanoma in vitro based on the Raman spectra of selected biochemicals. J Biomed Opt 2012;17(7):077003.

16. Mahadevan-Jansen A, Richards-Kortum RR. Raman spectroscopy for the detection of cancers and precancers. J Biomed Opt 1996;1(1):31-70.

17. Mahadevan-Jansen A, Mitchell MF, Ramanujam N, Malpica A, Thomsen S, Utzinger U, Richards-Kortum R. Near-infrared Raman spectroscopy for in vitro detection of cervical precancers. Photochem Photobiol 1998;68(1):123-132.
